# Supplementary material for: Survey on barriers to psychiatrists’ use of clozapine for young people in Scotland and suggestions for reducing these
Source: PLoS One. 2024 Jun 20;19(6):e0304996. doi: 10.1371/journal.pone.0304996 (PMC11189218; doi:10.1371/journal.pone.0304996)
Supplement: Attachment — Submitted filename: Yeni Microsoft Word Belgesi (2).docx [file pone.0304996.s001.docx]

This study is about the perspective of experts working with children and adolescents in Scotland on the use of clozapine.
