# Supplementary material for: Survey on barriers to psychiatrists’ use of clozapine for young people in Scotland and suggestions for reducing these
Source: PLoS One. 2024 Jun 20;19(6):e0304996. doi: 10.1371/journal.pone.0304996 (PMC11189218; doi:10.1371/journal.pone.0304996)
Supplement: Attachment — Submitted filename: Clozapine Survey Response to Reviewers Letter.docx [file pone.0304996.s002.docx]

PLOS One	Dr Graham Walker

1 May 2024	Clarice Pears Building

University of Glasgow

90 Byers Road

Glasgow

G12 8TB

[graham.walker5@nhs.scot](mailto:graham.walker5@nhs.scot)

07828561144

Dear Dr Balcioglu,

Thank you for providing your reviewers comments on our manuscript entitled “Survey on barriers to psychiatrists use of clozapine for young people in Scotland and suggestions for reducing these”.

I can confirm we have made the following recommended changes to our manuscript;

- We have updated our manuscript as per the PLOS One formatting guidelines.
- We have moved our ethics statement into the recruitment section of our methods as directed.
- We have removed figures from the main manuscript as directed.
- Our reference list has been updated with new included papers, focusing on relating our results to previous research.
- In response to reviewer 1, we have added several other references to clozapine related studies, including those considering a more global perspective.
- In response to reviewer 2, we have changed the order or the qualitative feedback tables to highest percentage at the top as advised. We have changed the corresponding themes/ subthemes in the text to this same order. We have also summarised the quotes to a more succinct level.
- We have uploaded our two figure files to the Preflight Analysis and Conversion Engine (PACE) digital diagnostic tool.

We hope that the following changes will make this article suitable for publication in your journal. If you would recommend any further changes, we are very happy to consider these also.

Yours sincerely,

Dr Graham Walker

BSc (Med Sci), MBChB, MRCPsych

Clinical Lecturer in Child and Adolescent Psychiatry, The University of Glasgow

ST4 CAMHS Psychiatry Registrar, West of Scotland
